# Supplementary material for: Myths, beliefs, and perceptions about COVID-19 in Ethiopia: A need to address information gaps and enable combating efforts
Source: PLoS One. 2020 Nov 30;15(11):e0243024. doi: 10.1371/journal.pone.0243024 (PMC7703946; doi:10.1371/journal.pone.0243024)
Supplement: S1 Questionnaire — (DOCX) [file pone.0243024.s001.docx]

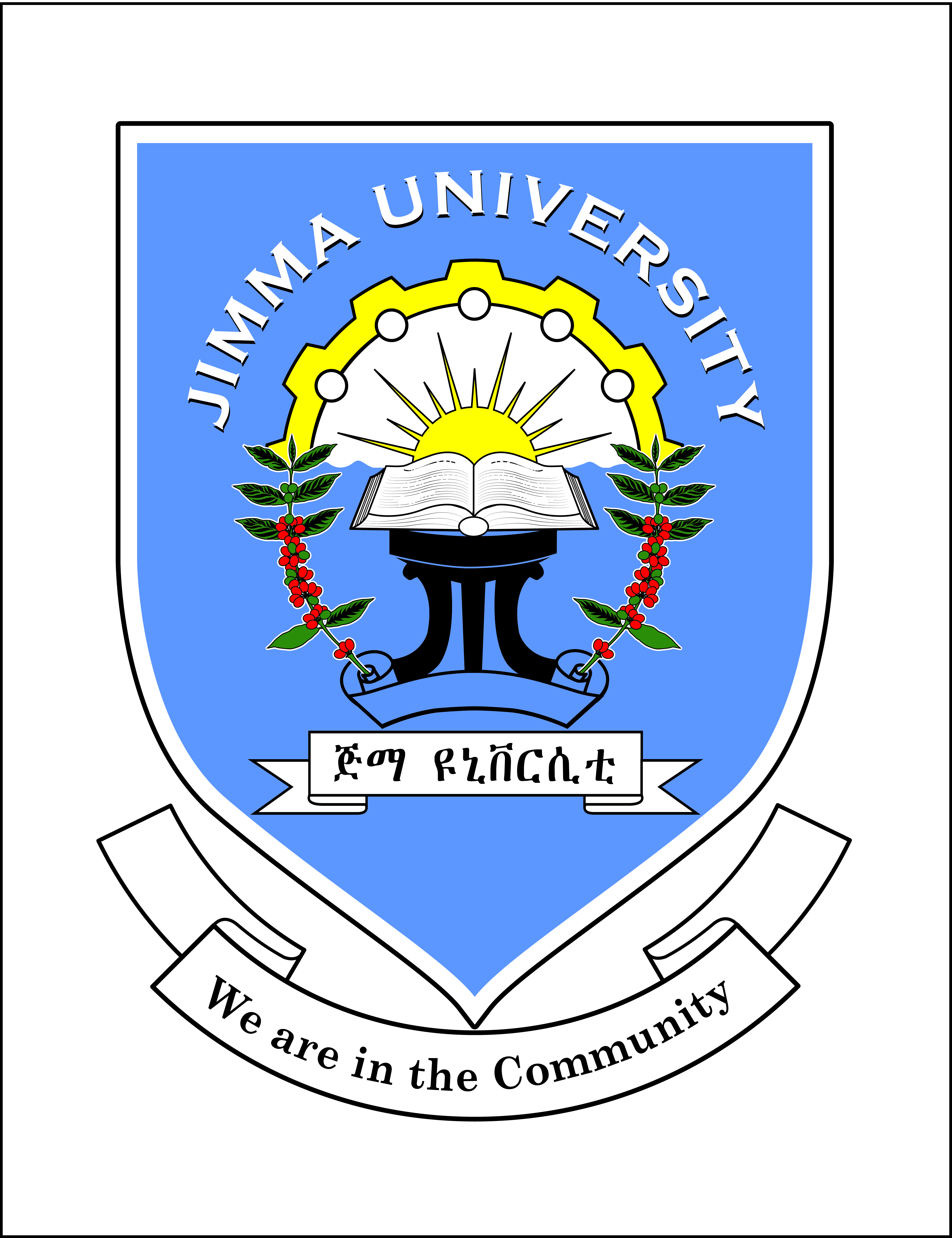


**Jimma University, Institute of Health**

**Questionnaire to assess community beliefs and perceptions towards the spread and control of COVID-19 in Ethiopia.**

Dear Sir/Madam,

Jimma University, Institute of Health conducting a nationwide rapid assessment to assess community perceptions and beliefs towards COVID-19 diseases among scholars who are supposed to take leading role in combating the spread- academic staffs, health professionals and other civil servants. The study would generate useful information and insight on scholars and community’s perceived awareness and extent of self-care practices and responses to communication messages. The finding will help to reshape and make further adaptations of ongoing efforts to prevent the spread of COVID-19 virus. Therefore, we kindly request your honest and kind response to this survey questionnaire. Indeed, your participation is voluntarily. But we highly value participation and contribution at this critical time. It may take 20-30 minutes to fill the question. Your responses will be completely anonymous. If you have any questions regarding this research, contact Zewdie Birhanu (PhD, phone:+251917025852, email: [zbkoricha@yahoo.com](mailto:zbkoricha@yahoo.com), Dr Diriba Fufa, email [diribafuf@gmail.com](mailto:diribafuf@gmail.com)), Mr. Yohannes Kebede (PhD Candidate, phone:+251913232040, email:yohanneskbd@gmail.com) or Prof. Argaw Ambelu (PhD, phone: +251911826218, email: aambelu@yahoo.com).

We would like to thank you for kindly consenting to participate in this survey.

Please click the link to access the questionnaire

**Part I: Background characteristics**

1. What is your age in completed years? ________________
2. What is your sex? A. Male ☐ B. Female ☐
3. What are your current educational qualifications?
4. ☐ Primary (grade 1-8)
5. ☐ High school (grade 9-12)
6. ☐ Diploma or Level I-IV
7. ☐ First Degree (BSc/BA)
8. ☐ Medical Doctor-GP
9. ☐ Residents (specify year of residency) _____________
10. ☐ Master degree
11. ☐ Ph.D
12. ☐ Clinical Specialty /sub-specialty
13. ☐ Other (specify)___________________________________
14. What is your current main occupational category in Jimma University?
15. ☐ Academic staffs /University lecturers
16. ☐ Administrative staff /
17. ☐ HospitalStaff /health professionals
18. ☐ Other offices,__________________________
19. Do you provide service in Jimma University Hospital?
20. ☐ Yes (specify your service category in Hospital):_________________________________
21. ☐ No
22. What is your current ma﻿rital status?
23. ☐ Single (unmarried)
24. ☐ Married
25. ☐ Divorced
26. ☐ Widowed
27. ☐ Engaged (in relationship)
28. What is your main occupation?
29. ☐ Merchant/shop
30. ☐ Teacher-elementary
31. ☐ teacher-high school
32. ☐ teacher University
33. ☐ Health care worker
34. ☐ Private work/business
35. ☐ Police/security
36. ☐ Military
37. ☐ Daily labouer
38. ☐ Farmer
39. ☐ Student
40. ☐ No job
41. ☐ Private company
42. ☐ Other (specify)_________________________
43. Where is your place of residence?
44. ☐ Urban
45. ☐ Rural
46. In which region of Ethiopia, are you currently living?
47. ☐ Addis Ababa
48. ☐ Oromia
49. ☐ Tigray
50. ☐ Ahmara
51. ☐ Somali
52. ☐ Affar
53. ☐ Benishangul Gumuz
54. ☐ Gambella
55. ☐ Harari
56. ☐ Dire Dawa
57. ☐ Southern nation nationalities and peoples
58. What is your religion affiliation?
59. ☐ Orthodox
60. ☐ Muslim
61. ☐ Protestant
62. ☐ Wakeffeta
63. ☐ Other (please specify)___________________
64. Which internet connection do you mostly use to get information about COVID-19?
65. ☐ Broadband-cable
66. ☐ Cellular-data(telle)
67. ☐ DSL (home private)
68. ☐ Wireless-Wifi
69. ☐ Do not use internet
70. On average, what is your monthly income in Ethiopian Birr? __________

**Part II: Communication sources**

1. What is your main source of information about COVID-19 disease?
2. ☐ Official websites
3. ☐ Television
4. ☐ Radio
5. ☐ Friends/Neighbors
6. ☐ Internet service (Wi-Fi)
7. ☐ Social media (Facebook, twitter, YouTube etc)
8. ☐ Health workers
9. Please write any call center you for information about COVID-19? _____________________
10. ☐ 8335
11. ☐ 6955
12. ☐ 6244
13. ☐ 6981
14. ☐ 6599
15. ☐ 6407
16. ☐ 6929
17. ☐ 6238
18. ☐ Other, specify________
19. Which source of information about COVID-19 do you most trust? (One or more options)
20. ☐ Government Medias (TV/Radio/newsletter etc)
21. ☐ Private Medias (TV/Radio/newsletter etc)
22. ☐ Religious leaders
23. ☐ Local source (announcement, posters, banners/brochures)
24. ☐ National source
25. ☐ International media
26. ☐ Social Medias (Facebook/whatsApp/Telegram/ etc)
27. ☐ Health Activists (Public figures/etc)
28. ☐ Health facilities/professionals
29. ☐ Other (please specify________________________

Part III: This section assesses how you perceive about community needs about gaps and communication topics that are urgently needed to Ethiopian Community. As you provide your response, please take a moment to refer to what you observe in your local community

1. People in your locality perceive that COVID-19 is a dangerous diseases?
   1. ☐ True
   2. ☐ False
   3. ☐ Don’t know
2. For people in your locality, who are at high risk of getting COVID-19? (Mark all those people think so)

1. □ Children (0-9) years old

2. □ Adolescents 10 to 15 years old

3. □ Youth (16-29 years) old

4. □ Adults (30-50 years) old

5. □ Elderly persons old

6. □ pregnant women

7. □ Health workers

8. □ People with underlying chronic illness (e.g:Diabetus, hypertension, cancer,etc)

9. ______________________________________

18. For people in your locality, who acquires severe form of COVID-19? (Mark all those people think so)

1. □ Children (0-9) years old

2. □ Adolescents 10 to 15 years old

3. □ Youth (16-29 years) old

4. □ Adults (30-50 years) old

5. □ Elderly persons old

6. □ pregnant women

7. □ Health workers

8. □ People with underlying chronic illness (e.g:Diabetus, hypertension, cancer,etc)

9. Other, __________________________________

19. As perceived by people in your locality, what are the norms that can exacerbate the spread of COVID-19?

1. □ People still live in a very crowned condition

2. □ People still use suffocated transportation means

3. □ People are still hugging/shaking hands as they greet

4. □ People cannot stay home for economic & other reasons

5. □ People don’t still adequately access water/sanitizers like alcohol

6. □ People don’t still access personal protective equipment like face masks

7. □ People with flu/similar symptoms are not well screened

8. □ I don’t know about what people perceive regarding norms that could spread COVID-19

9. Other_____________________________________

20. As perceived by people in your locality, what are the conditions that can lessen chance of attack by COVID-19?

1. □ They live in hot weather and that is not suitable for coronavirus

2. □ They live in setting that is far from coronavirus hot spot areas

3. □ They have adequate traditional protective medicines

4. □ They are religious enough

5. □ There are not reported cases yet in the their locality

6. □ They are engaging on major precautions very well

7. □ I don’t know about what people perceive regarding conditions that lessens the attach by COVID-19

8.Other _____________________________________

21. What do you think about what people in your locality would like to know about the COVID-19? (More options)

1. □ How to protect from the disease?

2. □ Symptoms of the new coronavirus disease

3. □ How it is transmitted

4. □ What to do if they have the symptoms

5. □ Most at risk groups

6. □ How to treat it

7. □ Other: _________________________

23. As perceived by people in your locality, COVID-19 is a stigmatized disease. 1. □ Yes 2 □ No

**Thank you for your participation and cooperation!**
